# A new method to assess perceived well-being among elderly people - a feasibility study

**DOI:** 10.1186/1471-2318-9-55

**Published:** 2009-12-03

**Authors:** Jori Reijula, Toni Rosendahl, Kari Reijula, Paula Roilas, Heikki Roilas, Raimo Sepponen

**Affiliations:** 1 Applied Electronics Group, Helsinki University of Technology, Otakaari 7A, Espoo, 02150, Finland; 2 Good Indoor Environment Theme, Finnish Institute of Occupational Health, Arinatie 3, Helsinki, 00370, Finland; 3 Tampere School of Public Health, University of Tampere, Tampere, 33014, Finland; 4 Department of Public Health, South Karelia Health Services, Lappeenranta, Finland

## Abstract

**Background:**

A simple but countable electronic device has been developed to gain reliable information on elderly patients' perceived well-being. The device has been tested and proven to be technically functional and countable. It was now tested in two care homes for the elderly and two private homes to evaluate if it provided solid information about the well-being of elderly persons. This report illustrates the practical usage of the device and shows its efficiency in gathering solid well-being information from the focus group.

**Methods:**

The test arrangement was carried out by assigning a group of volunteers (n = 10) in care homes for the elderly for two weeks. The time period was long enough to collect a sufficient amount of information to evaluate the perceived well-being of the test subjects. Perceived well-being was assessed by using a Con-Dis device and by filling out an attached questionnaire - RAI - at the same time. RAI consisted of questions concerning mood, pain and quality of life. A standardised RAVA questionnaire with 12 questions concerning test subject's health was also answered once during the two-week time period by each test subject. After the test period the data obtained by Con-Dis was compared with the findings collected using questionnaires.

**Results:**

A statistically significant correlation was found between perceived well-being (measured by Con-Dis) and questionnaire-based mood (r = 0,66, Pearson Correlation Coefficient) and quality of life (r = 0,68). No statistically significant correlation was found between perceived well-being and pain (r = 0,28). Technical functionality and feasibility of Con-Dis were good during the test period. Some problems arose because the test subjects were elderly and some in poor physical condition.

**Conclusion:**

On the basis of the collected results, the Con-Dis device presented information on the test subjects' perceived well-being that appeared to correlate with certain aspects of their health status. The test subjects' mood and quality of life but not pain had a statistically significant association with the perceived well-being level measured by Con-Dis.

## Background

The number of aging people in developed countries is rising. Meanwhile, the incidence of diabetes and cardiovascular diseases is also on the rise, along with the amount of work expected from healthcare professionals and the pressure exerted on them [1]. Voluntary self-monitoring should be emphasised in order to prevent the most common diseases from occurring [2]. The possibilities of health care technology are improving and perceived well-being, mood, pain, and quality of life measurement levels are efficient indicators of the onset of a serious disease. Thus, understanding patients' perceived well-being, mood, pain, and quality of life parameters is of great importance in order to predict the possible risk of their developing a serious disease or declining into a poor medical condition [3-9].

A simple but countable electronic device - Con-Dis - was developed to effortlessly provide information concerning a test subject's perceived well-being. The study design was to assess the practical usage and feasibility of the novel device among elderly persons living in care homes. The measurements were carried out during a two-week test period.

## Methods

### Study population

The present study was carried out in Lappeenranta, a city located 250 km east of Helsinki, Finland. A total of ten elderly test subjects (7 women) aged 63-89 (mean 78) were randomly selected for the study. The duration of living in elderly care home varied from four weeks to five years. Two test subjects lived in a municipally owned care home for the elderly (A) and six of the test subjects came from a care home for the elderly (B) that was owned by a private foundation. The other two test subjects lived at home.

The first care home for the elderly (A) was staffed by day and night nurses. Both test subjects had a single room with its own kitchen and bathroom. However, only one test subject used the nursing service and had a walking support device to ease her movement around the apartment. The second home (B) did not have nurse assistance. The rooms in care home B were either single or double apartments with normal living conditions. Two test subjects also lived at home without assistance.

### Con-Dis device

A detailed description of the Con-Dis device has been provided elsewhere [10]. Briefly, Con-Dis is a monitoring system that records patients' evaluations of their perceived levels of well-being and stores the information for later access. Con-Dis consists of three buttons - happy, neutral, and unhappy - each illustrating the patient's perceived well-being.

Perceived well-being in the present study is only based on the subjective experience of the test subjects. To report perceived well-being, each test subject was asked to assess the present perception of their physical and mental condition.

### RAI and RAVA

The device was tested in the present field study by using a modified questionnaire composed of three RAI (Resident Assessment Instrument) questions as a gold standard [11,12]. A standardised RAI questionnaire comprises 160 questions. These three questions (mood, pain, and quality of life) were selected because they widely measure the patients' daily routines. Each of the test subjects reported on their well-being three times per day. Each morning, afternoon, and evening they answered the three RAI questions about their perceived mood, pain, and quality of life. They were supposed to report if they felt their mood to be depressed, normal, or good by putting a cross in a box corresponding to one of the three. The same applied to pain: whether the test subject felt no pain at all, a little pain, or constant, bothering pain. The test subjects' experienced quality of life was also measured similarly: whether they weren't feeling good, they were feeling okay, or they were feeling good. After reporting the RAI parameters, the test subjects reported their perceived well-being by pressing one of three Con-Dis buttons (happy, neutral, or unhappy - explained above). The collected results were gathered and analysed. Thus, during the two-week time period each person was supposed to use the Con-Dis device a total of 42 times and answer 126 RAI questions altogether.

The RAVA (Rajala-Vaissi) index was assessed by using a standardised questionnaire with 12 specific questions about the test subjects' health [13,14]. The questions evaluated their sight, hearing ability, speaking ability, mobility, urine, stools, eating, medicine usage, ability to dress and wash, memory functionality, and psyche. As a result, the test subjects received a RAVA index score (range 1.29-4.23 or grading 1-6) of their overall health. A low RAVA index score means good overall health and a high score means poor overall health [15]. The RAVA index is an older and simpler questionnaire than RAI. It was used for comparison, because the tests have minor differences [15].

### Test protocol

Overall, 10 test subjects in two care homes for the elderly and private homes reported their perceived well-being using the Con-Dis device and answering the RAI questionnaire study three times per day (each morning, noon, and late evening) over the two-week test period. This adds up to a total of 42 perceived well-being evaluations per test subject. In addition, the test subjects answered 12 RAVA questions about their health. Before the test period, the test subjects were trained by a nurse to use the Con-Dis device and to fill out the RAI questionnaire. The test subjects were asked to push the buttons in the manner described below.

The "happy face" button should be pressed at the given time intervals if the test subjects assume their well-being to be better than their average. The "happy face" represents a situation in which the test subject feels no abnormal pain, their mental situation does not include depression, and their physical condition is above average at the moment.

The "neutral face" button should be pressed if the test subjects assume their well-being is average. The "neutral face" depicts a situation of a condition of stable well-being condition for the test subject. Mild but not harmful pain can be accepted and their mental situation and physical condition are seen as average.

The "unhappy face" button should be pressed at the given time intervals if the test subjects assume their well-being is worse than average. The "unhappy face" represents a situation in which the test subject feels moderate or severe pain. The person is suffering from depression and their physical condition may be notably below average.

The test subjects were asked to contact a selected researcher or nurse if they needed further instructions or assistance in operating the Con-Dis device. The data were collected from the Con-Dis device by using a (SD) memory card. The memory card included a simple utility program that displays the well-being and mood measurements when inserted into a PC.

The present study has been approved by the ethical committee of the Pirkanmaa Hospital District, Tampere, Finland.

### Statistical methods

**Figure 1:**
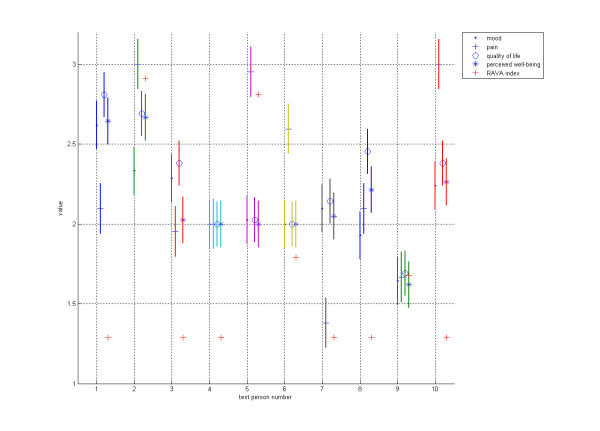
**Test results 1**. Data collected from 10 test subjects during a 2-week survey using the RAI questionnaire (mood, pain, quality of life), Con-Dis device (perceived well-being) and RAVA index (see the text). Each test subject is shown on the x-axis (subjects numbered 1-10) and their reported well-being parameters (as mean values), along with their probability errors, are shown on the y-axis. Values on the y-axis are between 1 (unhappy) and 3 (happy).

**Figure 2:**
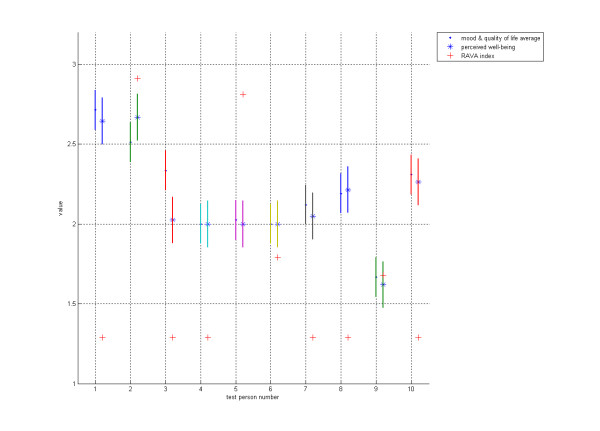
**Test results 2**. The average of mood and quality of life, along with the Con-Dis device's perceived well-being information from 10 persons, during the two-week test period are shown. Each test subject is shown on the x-axis (subjects numbered 1-10) and their reported well-being parameters, along with their probability errors, are shown on the y-axis. Values on the y-axis are between 1 (unhappy) and 3 (happy).

The probability errors in Figure 1 and Figure 2 were measured using the Matlab "Anova" function. Statistical differences in the levels between the groups were tested using the SAS 9.1 program and Pearson Correlation Coefficients.

## Results

The test protocol was carried out successfully and all 10 devices functioned well throughout the test period. No problems concerning the technical functionality or feasibility of Con-Dis were found. Some test subjects did not remember or otherwise were unable to report their well-being using RAI questions and Con-Dis at all the required times. However, each test subject reported most of the required information, with over 90% of the total number of questions being answered. One of the test subjects accidentally managed to pull the Con-Dis device's power cord out of the socket but the measurement was not disrupted. Some test subjects unintentionally pressed the device multiple times. Some misinterpretations of the instructions on how to use the Con-Dis device were noticed. For example, test subject number 4 answered "neutral" 124 times out of the total of 126, which can be seen as distorted error margins in Figure 1. Test subject number 9 also had seemingly small error margins (Figure 1), although the results varied more. All in all, most test subjects understood the instructions correctly and their answers appeared to be rational. The difference between test subjects having "good" days and those having "bad" days could also be observed from their answers (Figure 1) because some appeared to be happier than others overall. The RAVA index scores were also calculated, but they did not have a close statistical correlation with either the RAI or the Con-Dis answers (Figure 1).

On the basis of the collected results from the ten test subjects and their 420 individual markings during the two-week period, the perceived welfare of a test subject - measured with Con-Dis - closely correlated with the test subject's mood (r = 0.66, Pearson Correlation Coefficient) and quality of life (r = 0.68). On the other hand, the patient's perceived pain level differed significantly from their mood (r = 0.21), quality of life (r = 0.28), and perceived well-being (r = 0.37) (Table 1). The association and difference between the mean values in all of the above-mentioned parameters were statistically very significant (p < 0,0001).

**Table 1:** Subjects' r-values.

Parameter	Mood	Pain	Quality of life	Perceived well- being
**Mood**		0.210	0.591	0.658

**Pain**	0.210		0.281	0.366

**Quality of Life**	0.591	0.281		0.682

**Perceived well- being**	0.658	0.366	0.682	

Table 1 shows a chart of the test subjects' Pearson Correlation Coefficients (r-values) between mood, pain, quality of life, and perceived well-being. For each of the cases, the association and difference between the mean values in all of the above-mentioned parameters were also measured and were statistically very significant (p < 0,0001).

In Figure 1 the individual results of each test subject show that there was a close correlation between perceived well-being, mood, and quality of life. On the other hand, among most of the test subjects there was a difference between the findings of pain and perceived well-being but also with mood and quality of life.

Figure 2 illustrates the findings further. The test subjects are numbered along the x-axis and the averaged parameters of their mood and quality of life, along with the perceived well-being parameter, are shown on the y-axis. The figure shows a close statistical correlation between the average for their mood and quality of life and the perceived level of well-being.

## Discussion

The present study, focusing on the usability and feasibility of the Con-Dis device among elderly test subjects, is the first of its kind in assessing a simple electronic device measuring perceived well-being. This information will become very important in the near future because of the need to closely monitor old people at home or in care homes for the elderly rather than in hospitals [16,17]. Medical professionals need to monitor certain parameters of their health condition in order to be able to respond if changes occur in vital physiological functions [18,19]. Therefore, health technology has to be improved and novel devices have to be invented and tested for monitoring purposes.

The Con-Dis device was invented recently and the methodology has already been published elsewhere earlier [10]. The Con-Dis device functioned well in the present field study. The results of the field study among ten elderly test subjects were seen to be countable and the sample size was considered to be sufficient to present feasibility study of the novel device. Even though some test subjects did not answer every question required, they still answered over 90% of the total number of questions, which was considered to be enough to validate the study.

Possible limitations of the present study were the sample size (only 10 test subjects) and the two-week time frame. However, we think that the present data justifies the interpretation concerning feasibility of the Con-Dis device to be used among elderly people.

The test appeared to have one outlier - a test subject (number four), who did not understand the instructions that were given or was otherwise unwilling to express his mood by pressing a button other than "neutral" (test subject number four in Figure 1). It might also be possible that he did perceive his mood to be stable and neutral during the two-week time period. On the other hand, he might have been a person not accustomed to self-observation and recognition of his mood, pain and perceived well-being fluctuations. However, his answers did not noticeably alter the test results and were thus accepted for inclusion in the study.

Pain differed remarkably from the other parameters measured. This suggests that Con-Dis does not take pain into account specifically when measuring the perceived well-being of a test subject (r = 0,37). However, the perceived level of well-being (measured with Con-Dis) was greatly affected by the test subject's mood (r = 0,66) and perception of their quality of life (r = 0,68). We emphasize, however, that the basic objective of the present study was not to elucidate the complex phenomena of perceived well-being related to specifically pain.

In an earlier study, pain had a correlation with patients' mood and anxiety among chronic pain patients [20]. According to another study on chronic (non-malignant) pain patients, statistically significant but modest correlations were found between the severity of their pain and their health-related quality of life [21]. Psychological and social well-being was closely correlated as well [21]. The present study, however, does not agree that pain has a close statistical correlation with either mood (r = 0,21) or quality of life (r = 0,28). The difference between the results of previous studies [20,21] and our findings may arise from the different focus groups. Chronic pain patients' mood and quality of life may be significantly more affected by pain than that of the non-chronic test subjects used in the present study.

Perceived well-being has earlier been assessed by using RAI and RAVA [11-14]. According to the previous studies RAVA and, especially, the newer and more complicated RAI questionnaire provided information on a patient's health status, whether a patient is in a healthy physical and mental condition or not [11-14].

The present study appears to prove that Con-Dis can give adequate information on a test subject's perceived well-being, because the average of mood and quality of life is very closely correlated with the test subject's perceived well-being, recorded using Con-Dis (Figure 2). Con-Dis is remarkably faster and easier to use than a multi-parameter RAI index - which is seen as too laborious for the monitoring of daily well-being [22] - and could therefore be used as a monitoring device for the elderly and handicapped. According to our preliminary experience, Con-Dis could also be used in non-health assessments in social and health care services such as evaluating quality of services.

## Conclusion

On the basis of the collected results, the Con-Dis device presented information on the test subjects' perceived well-being that appeared to correlate with their health status. The test subjects' mood and quality of life but not pain correlated closely to the perceived well-being level measured by Con-Dis.

Con-Dis is also seen as a countable device to measure an elderly person's well-being. Old age does not render the device unusable. According to the present data we suggest that the Con-Dis device can be used in assessing the mood and quality of life, but not the severity of pain, of elderly people.

## Competing interests

A patent application for "Con-Dis" device is currently pending.

## Authors' contributions

JR was responsible for collecting the test information, processing the information, and writing the article. TR was responsible for designing and building the Con-Dis device and providing help with the Con-Dis device. PR was responsible for organising the test in Lappeenranta and collecting the data and test information. HR and KR were responsible for providing help in planning the project and with medical questions. RS was the director of the project and came up with the idea of Con-Dis. All authors read and approved the final manuscript.

## Pre-publication history

The pre-publication history for this paper can be accessed here:

http://www.biomedcentral.com/1471-2318/9/55/prepub
